# Parallel Colorimetric Quantification of Choline and Phosphocholine as a Method for Studying Choline Kinase Activity in Complex Mixtures

**DOI:** 10.3390/antibiotics7010024

**Published:** 2018-03-17

**Authors:** Tahl Zimmerman, Salam A. Ibrahim

**Affiliations:** Food Microbiology and Biotechnology Laboratory, Food and Nutritional Sciences Program, North Carolina A & T State University, 1601 East Market Street, Greensboro, NC 27411, USA; ibrah001@ncat.edu

**Keywords:** choline kinase, *S. pneumonia*, choline, phosphocholine, colorimetric methods, enzymes, gram-positive bacteria

## Abstract

Choline kinase (Chok) is an enzyme found in eukaryotes and Gram-positive bacteria. Chok catalyzes the production of phosphocholine from choline and ATP. This enzyme has been validated as a drug target in *Streptococcus pneumonia*, but the role Chok enzymatic activity plays in bacterial cell growth and division is not well understood. Phosphocholine production by Chok and its attenuation by inhibitors in the context of complex samples such as cell extracts can currently be quantified by several methods. These include choline depletion measurements, radioactive methods, mass-spectrometry, and nuclear magnetic resonance. The first does not measure phosphocholine directly, the second requires elaborate safety procedures, and the third and fourth require significant capital investments and technical expertise. For these reasons, a less expensive, higher throughput, more easily accessible assay is needed to facilitate further study in Gram-positive Choks. Here, we present the development of a triiodide/activated charcoal/molybdenum blue system for detecting and quantifying choline and phosphocholine in parallel. We demonstrate that this system can reliably quantify changes in choline and phosphocholine concentrations over time in Chok enzymatic assays using cell extracts as the source of the enzyme. This is an easily accessible, convenient, robust, and economical method for studying Chok activity in complex samples. The triiodide/activated charcoal/molybdenum blue system opens new doors into the study choline kinase in Gram-positive pathogens.

## 1. Introduction

Choline kinase (Chok) is an enzyme that catalyzes the production of phosphocholine (PCho) from choline (Cho) and ATP [1]. Chok enzymes play a key role in cell growth and division in eukaryotic cells [2] and are also oncogenic drug targets for cancer cells [3] as well as for parasites such as *Plasmodium falciparum* [4]. However, the role Chok plays in bacterial cell division and growth is less clear, although it is known to be involved in the pathway leading to the production of lipoteichoic acid (LTA) and cell wall teichoic acid (CTA) [5,6]. *Streptococcus pneumoniae* is a pathogen known to express Chok [7]. Experimentally unconfirmed sequence predictions suggest that other Gram-positive pathogens, such as *Stapholooccus aureus*, *Bacillus subtilis*, *Clostridium perfringens*, and *Clostridium botulinum* also produce Chok isoforms. Importantly, the choline kinase of *Streptococcus pneumoniae* (sChok) has recently been established as a drug target [8], and inhibiting sChok was found to effectively slow cell growth and division in this species. However, Chok activity is confirmed in *S. pneumoniae* alone and has not yet been well characterized in this species of bacteria. Consequently, further studies on the enzymatic activity of sChok as well as other bacterial Choks are warranted, as is the development of tools designed to facilitate these studies.

When adding a cell extract containing Chok to a solution of ATP and Cho, the resulting activity generates a complex mixture containing choline, ATP, PCho and Cho. The rate of the PCho production can be inferred indirectly from the rate of Cho consumption through colorimetric methods involving triiodide precipitation [1,9] or Cho conversion to betaine aldehyde by choline oxidase [10]. Phosphocholine can be directly detected by utilizing ^14^C Cho substrate in an enzymatic reaction, followed by a thin layer chromatography step to separate the reactants from products, followed by detection using a phosphoimager [4]. Organic chemistry fractionation techniques can also be used to separate the reactants from the products [11]. Radioactive methods require added safety procedures because, if ingested, ^14^C Cho can accumulate in organs, exposing cells directly to emissions, causing DNA damage. For example, a separate space in the laboratory is necessary for dispensing radioactive reagents, as well as shielding. Double gloves need to be used when handling isotopes, and workspaces must be monitored regularly with Geiger counters. In addition, accessibility is limited because a specialized infrastructure is required to monitor individuals for contamination using dosage monitors and urine testing [12,13]. While highly sensitive, radioactive methods for measuring PCho lead to only relative, rather than absolute quantities. Much safer and quantitative methods for measuring PCho include mass spectrometry [8], and nuclear magnetic resonance [14]. However, these techniques are not accessible or economical because they require specialized training and significant capital investments. Moreover, all the non-colorimetric methods for measuring PCho or choline are time consuming because the number of samples that can be tested in parallel is limited. To study choline kinase function in greater detail, a method that is accessible and economical is needed to quantify the production of PCho. In addition, a convenient, more quantitative benchtop alternative to radioisotope methods is needed that requires fewer safety procedures. We present here the development of an easy-to-implement colorimetric method to detect and quantify both Cho and PCho using absorbance at wavelengths in the visible range.

## 2. Results

### Development of the Detection Method

**Figure 1:**
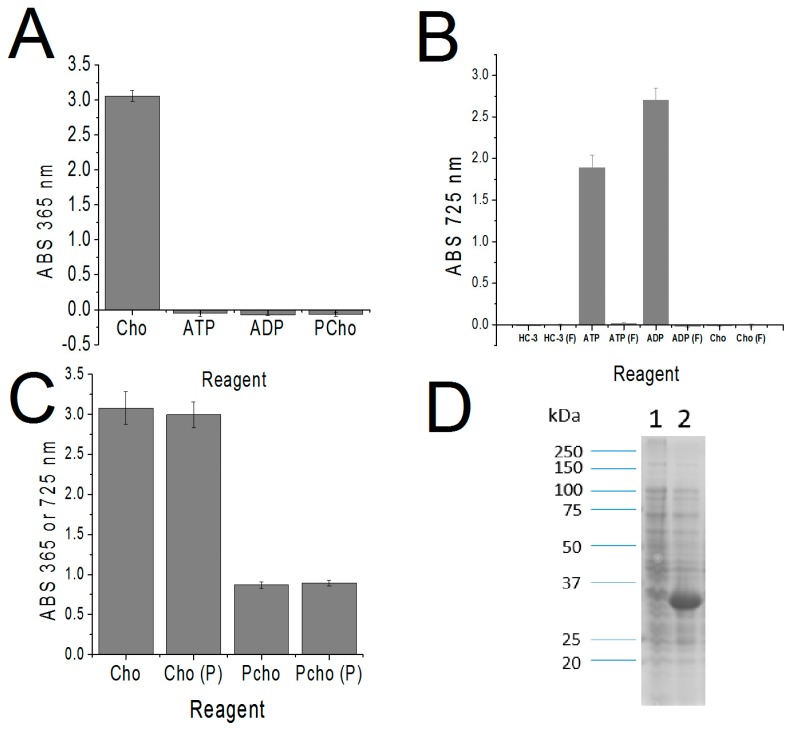
Validation of the colorimetric method. A 1 mM concentration of each reagent was assayed, except for 2.7 mM of HC-3. (**A**) Triiodide reactions with each component of the Chok reaction: choline (Cho), ATP, and phopshocholine (PCho). (**B**) Analysis of MBD absorbance measurements after processing of each compound with triiodide step alone and the combined steps of triiodide and charcoal (marked with an (F)). (**C**) Absorbance values of PCho and Cho samples detected with and without processing (P). (**D**) SDS-PAGE of extracts of uninduced (1) and induced (2) BL21 (DE3) cells transformed with *S. pneumoniae LicA*, the gene coding for sChok.

In our initial search of viable methods for the colorimetric detection of PCho, we encountered a past study which demonstrated that PCho could be precipitated as a complex with a molybdenum blue dye (MBD) with 90% efficiency [15], resuspended in an acetone/HCl solution, and then quantified by absorbance at 725 nm. The lower limit of sensitivity of the MBD was 100 μM. However, this dye was also found to precipitate Cho (data not shown), ATP (Figure 1B), and ADP (Figure 1B). Due to its promiscuity, use of MBD alone was not deemed sufficient to reliably quantify PCho. To employ the MBD dye to detect PCho, the contaminants Cho, ATP, and ADP had to first be filtered out of the solution.

To accomplish the ADP/ATP filtration step, activated carbon prepared in 0.1 N HCl was used as a highly efficient nucleotide filter [16]. Using this method ATP and ADP were filtered out to levels undetected by the MBD (see Figure 1B).

**Figure 2:**
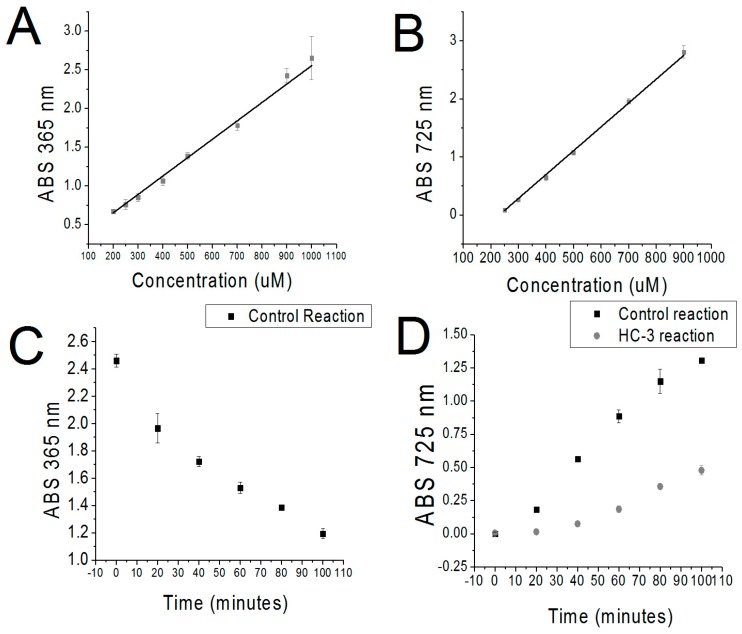
Standard concentration vs absorbance curves of Cho and PCho and colorimetric absorbance at different time points in an sChok enzymatic assay. (**A**) Standard curve of [Cho] vs absorbance derived from known quantities of Cho. (**B**) Standard curve of [PCho] vs. absorbance derived from known quantities of PCho. (**C**) Absorbance changes over time derived triiodide analysis of an sChok enzymatic assay (Control Reaction). (**D**) Absorbance changes over time derived from MBD analysis of an sChok enzymatic assay with (HC-3 reaction) and without (Control Reaction) sChok inhibitor HC-3.

Meanwhile, Cho was filtered out using a method adapted from Appleton et al. [9]. This method employed a triiodide solution to convert Cho to choline iodide, which was then precipitated by centrifugation, leaving only PCho in solution. Significantly, the triiodide method could be used to quantify Cho, because, as reported, the amount of precipitate was proportional to the concentration of Cho in solution. After resuspension of the choline iodide in ethylene dichloride, absorbance was measured at 365 nm [9]. A standard curve correlating known amounts of Cho with the measure of absorbance was constructed for use in elucidating unknown quantities of Cho (see Figure 2A). This method was sensitive to a lower limit of 50 μM.

Therefore, the complete PCho/Cho detection method for ChoK enzymatic reactions had three principle steps: (1) filtration of ATP/ADP by activated charcoal; (2) Cho quantification (and removal) using a triiodide solution; and (3) PCho quantification using an MBD solution.

However, three important questions had to be answered to determine if this method was robust: (1) Did triiodide cross-react with any other component of the reaction (PCho, ADP, ATP)? (2) Did the initial activated charcoal and triiodide steps filter out the confounding agents (Cho/ADP/ATP) to levels undetectable by the MBD dye so that only PCho was detected? (3) Was there a detectable loss of Cho or PCho during the filtration steps which could confound the measurements?

As a first step, we determined that the triiodide precipitated Cho but did not precipitate ATP, ADP, or PCho (Figure 1A). As seen in Figure 1B, the combined activated charcoal and triiodide steps removed the sChok inhibitor Hemicholinium-3 (HC-3) and Cho to levels that were undetectable by the MBD agent. Meanwhile, ATP and ADP were efficiently filtered by the activated charcoal step alone ((Figure 1B, ATP (F) and ADP (F)). As a result, all confounding agents were removed in the filtration steps leaving PCho alone to be detected by the MBD. In addition, the filtration steps did not lead to meaningful losses of Cho or PCho (Figure 1C).

The primary objective of this study was to develop a colorimetric method for detecting the amount of PCho generated from Cho and ATP using a complex protein mixture as the Chok source. As a model, we selected the sChok expressed in *E. coli* [7]. The *S. pneumoniae LicA* gene overexpressed well in BL21(DE3) cells (Figure 1D). The SDS-Page analysis clearly showed a strong band at around the expected size (35.5 kDa) in the induced sample, indicating that Cho kinase was overproduced against a background of endogenous proteins.

A time course of enzymatic activity was performed over a period of 100 min using cell extracts of sChok overexpressed in BL21 (DE3). Samples were taken every 20 min. In addition, a second time course was performed using HC-3, a known inhibitor of sChok [8], for comparison purposes. A second objective of this study was to demonstrate that this system was a viable method for drug screening against bacterial Chok enzymes. A linear reduction of Cho was observed over time in parallel with an observed increase in PCho (see Figure 2C,D). Quantification of Cho and PCho demonstrated that the two separate measurements corresponded with minor deviations when the concentration of Pcho was inferred from the concentration of Cho (see Table 1, [PCho] vs inverse [Cho]). Cho quantification was confounded in the case of the reaction containing HC-3 because this compound precipitated alongside Cho in the triiodide step. Nevertheless, HC-3 was no longer present in quantities that interfered with the MBD step (See Figure 1B). This meant that PCho could be reliably measured even when HC-3 was included in an enzymatic reaction. However, Cho could not be measured in the case HC-3 was added to the reaction. As a final validation step, the concentration of Cho and PCho found at 60, 80, and 100 min time points was measured by mass spectrometry (MS). MS values were found to be comparable to the colorimetric values. The results of the MS assays also confirmed that the catalysis of choline was inhibited in the presence of HC-3 (Table 1). Consequently, overall, this colorimetric method could be considered to be validated.

## 3. Discussion

We have presented here a combined MBD/activated charcoal/triiodide method for the simultaneous quantification of Cho and PCho for use in measuring changes in Cho and PCho concentration during choline kinase enzymatic assays.

The Cho and PCho quantification overlapped in the control enzymatic reaction. This meant that the method was self-verifying. In addition, this dual quantification method is flexible enough to overcome sensitivity limitations. Since the initial amount of Cho in an enzymatic reaction is fixed, the amount of Cho measured at any time point is inversely related to the amount of PCho. Therefore, even when the MBD cannot quantify PCho directly because the concentration of PCho does not meet the sensitivity threshold for this dye, an indirect reading is still possible using the triiodide, and vice versa. Most importantly, this system is redundant and, as such, measurements can be made in a way that prevents the confounding effects of inhibitors. If a tested inhibitor interferes with one set of measurements, as in the case of HC-3 and Cho, by definition this inhibitor cannot interfere with the second set of measurements because the inhibitor will have precipitated in only one of the steps. That is, the interfering molecule is precipitated either with the MBD or the triiodide, but not both. In addition, the activated charcoal itself removed a large part of the HC-3 (data not shown). It is likely that many other aromatic molecules could be filtered out to some degree. In our case, the amount of HC-3 was simply too elevated (2.7 mM) for the activated charcoal to handle alone, and the rest was precipitated by the triiodide. However, the activated charcoal alone may be sufficient to remove aromatic inhibitors that are effective in the μM range. Therefore, this colorimetric system could feasibly be used for medium throughput screening of choline kinase inhibitors.

This is the first known colorimetric method for quantifying phosphocholine. We have demonstrated here a robust, highly accessible, and economical method for the quantification of both Cho and PCho. In addition, we have shown that this method can be used to monitor the consumption of Cho in parallel with the production of PCho in enzymatic reactions using cell extracts as the source of the ChoK enzyme. Using this method, ChoK enzyme inhibition can be monitored and characterized and can be studied as part of a complex mixture without prior enrichment. This colorimetric technique requires only a few simple reagents and three pieces of equipment that are available in most biochemistry laboratories: a scale for measuring reagents, a centrifuge to remove precipitants, and a spectrophotometer to carry out measurements. This method is optimized for use in quantifying the generation of PCho enzymatic reactions using cell extracts as the enzyme source. The development of this method also supports exploration into the role of Choks in Gram-positive strains as well as bacterial pathogens already known to express Chok and provides a simple method for medium-throughput screening for potential inhibitors of bacterial ChoKs. This result is accomplished without resorting to the use of methods that require a high capital investment or specialized training. Radioactive methods are highly sensitive, reaching the picomole range [11]. Our method can be used to detect choline and phosphocholine at 50 μM and 200 μM, respectively, and is therefore far less sensitive. Nevertheless, this colorimetric method is more quantitative and can be used without the need for the elaborate safety procedures required for the use of radioisotopes. Use of this technique will thus facilitate clarification of the role Chok plays in Gram-positive bacteria.

## 4. Materials and Methods

All chemical reagents were purchased from Sigma-Aldrich, unless otherwise noted.

### 4.1. Preparation of Enzyme Extracts

The *S. pneumoniae* LicA gene expressing sChok had been previously cloned into the pET28a plasmid [7]. This construct was generously provided to us by the group of Dr. Yuxing Chen. The plasmid was transformed into BL21(DE3) cells (New England Biolabs). These cells were then used to inoculate 10 mL of Luria Broth (LB) which was incubated overnight at 37 °C. The next morning, a 10 mL of fresh LB was inoculated to 2% with the overnight culture and then incubated at 37 °C until an O.D._600_ of 0.6 was reached. The culture was cooled on ice, and IPTG was added to a final concentration of 1 mM to induce production of sChok. The culture was then incubated overnight at room temperature after which it was centrifuged for 10 min at 3500 *g*, resuspended in 1 mL 100 mM Tris pH 8 and transferred to an Eppendorf tube. Three mg of Glasperlen beads (Sartorius Stedim) were added to the resuspension and lysis was performed using a Bead-Beater 16 (Biospec products). The lysate was centrifuged at 20,000 *g* for 30 min at 4 °C. The supernatant was removed, aliquoted into Eppendorf tubes, and stored at −20 °C. An aliquot was defrosted on ice when needed for use in an enzymatic reaction.

### 4.2. sChok Enzymatic Reaction

Four microliters of Chok enzyme extract were added per 15 mL reaction buffer (RB: 100 mM Tris, 10 mM MgCl_2_, 1 mM Cho, 1 mM ATP) with or without 2.7 mM HC-3, and the reactions were incubated in a water bath at 37 °C for 100 min. One-milliliter samples were removed in duplicate every 20 min and placed in Eppendorf tubes on ice. The samples were immediately heated to 95 °C in a heat block for 3 min to stop the reaction. Samples were then placed on ice again for a minimum of 10 min.

### 4.3. Activated Charcoal Filtration of ATP

Activated charcoal was suspended in 0.1 N HCl for a total of 2.5 g/50 mL and then mixed by inversion and centrifuged at 4000 *g* for 20 min at 4 °C. The supernatant was removed and the charcoal pellet was resuspended in fresh 0.1 N HCl. This sequence was repeated 3 times, and the charcoal was resuspended in 50 mL 0.1 N HCl/2.5 g. The suspension was stored at 4 °C until needed.

Three-hundred microliters of this suspension were added to the enzymatic reaction sample and mixed by inversion at 1 min intervals for 10 min and then centrifuged in tubes at 20,000 *g* 4 °C for 1 h to remove the charcoal and denatured protein. One milliliter of supernatant was transferred to a clean 1.5 milliliter Eppendorf tube. The Cho in these samples was then quantified.

### 4.4. Triiodide Quantification/Removal of Cho

A solution of potassium triiodide was prepared using the following reagents (per 100 mL deionized water): 15.7 g of reagent grade iodine and 20 g of reagent grade potassium iodide. The solution was stored at 4 °C until immediately before use.

Four-hundred microliters of triiodide solution were added to each charcoal supernatant sample after which the mixture was immediately placed on ice for 1 h. The samples were then centrifuged at room temperature for 15 min at 20,000 *g*. One milliliter of each sample in triiodide solution was then set aside in fresh Eppendorf tubes for the subsequent PCho quantification. The remainder of the supernatant was discarded without disturbing the pellet which exhibited a dark red color. Because chlorine iodide decays quickly [9], 1 mL 1,2-Dichloroethane, was added immediately. The pellets were then dissolved by vortexing. Some charcoal fines were occasionally left over from the previous step. However, these did not dissolve and were left for 1 min to settle to the bottom of the tube before continuing with the procedure. As previously reported, the residual triiodide solution did not interfere with subsequent measurements. Forty microliters of each sample were aliquoted into wells of Greiner Bio-one CellStar^®^ U-bottom 96 well-plates. One hundred sixty microliters 1,2-Dichloroethane were then added to each sample-containing well. Two hundred microliters 1,2-Dichloroethane were added to an empty well in the plate and used as the blank. Absorbance was measured at 365 nm in a BioTek Synergy HT microplate reader.

### 4.5. MBD Quantification of PCho

Molybdenum blue dye (MBD) was prepared fresh daily two hours before use. One and two-tenths of a gram of Phosphomolybdic acid hydrate (Sigma-Aldrich, St. Louis, MO, USA) and 0.2 g Stannous chloride (Fisher Scientific, Hampton, NH, USA) were dissolved in 2.5 N HCl by vortexing for 1 min. Ten milliliters of deionized water were added, and the mixture was vortexed for 1 more min. The resulting solution was then filtered using a 0.2 M GPF/CA membrane non-sterile syringe filter (Phenomenex, Torrance, CA, USA).

Four hundred microliters of MBD were added to 1 mL enzymatic assay samples in triiodide. The samples were placed on ice for two hours and then centrifuged at 20,000 *g* for 3 min at room temperature. The pellets had a dark blue color. The supernatants were discarded and 1 mL of a 1:1 solution of 2.5 N HCl:acetone was added to the pellet resulting in a blue solution whose intensity increased with concentration. Two hundred microliters of each resuspension were aliquoted onto a well in a Greiner Bio-one CellStar^®^ U-bottom 96-well plate. Absorbance readings were immediately made at 725 nm using a BioTek Synergy HT microplate reader.

### 4.6. Mass Spectrometry

Enzymatic assay samples were spiked with stable labeled internal standards of all the analytes and extracted using a modified method from [17]. Samples were extracted with 4 volumes of methanol/chloroform (2:1, *v*/*v*) containing internal standards. The mixture was vortexed and stored at 4 °C for 2–24 h. Samples were centrifuged, supernatants were transferred to microcentrifuge tubes, and the pellets were re-extracted with methanol/chloroform/water (2:1:0.8, *v*/*v*/*v*). After vortexing and centrifugation, the supernatants were collected and combined with the first supernatant. Water and chloroform were added to induce phase separation. After centrifugation, 50 µL of the aqueous phase (containing choline and phosphocholine) were transferred to HPLC vials containing 100 µL acetonitrile for instrumental analysis. A series of standards of known concentration containing the corresponding internal standards were prepared and treated identically to the samples.

Quantification of the analytes was performed using liquid chromatography-stable isotope dilution-multiple reaction monitoring mass spectrometry (LC-SID-MRM/MS). Chromatographic separations were performed on an Acquity HILIC 1.6 µm 2.1 × 50 mm column (Waters Corp, Milford, MA, USA) using a Waters ACQUITY UPLC system. The column was warmed to 40 °C, and the flow rate maintained at 0.37 mL/min. The mobile phases were: A-100% water with 0.1% formic acid, and B-90% acetonitrile/10% water with 10 mM ammonium formate and 0.125% formic acid.

The gradient was 0% A/100% B, to 40% A/60% B to 2.5 min, to 70% A/30% B to 4.5 min, and 0% A/100% B to 5 min (total run = 5 min). The analytes and their corresponding isotopes were monitored on a Waters TQ detector using characteristic precursor-product ion transitions indicated in Table 2. Concentrations of each analyte in the samples were determined using the peak area ratio of the analyte to its isotope (response), and read off a standard curve of response values versus standard concentration for each analyte.

## Figures and Tables

**Table 1:** Comparisons between PCho and Cho colorimetric measurements, as well as the PCho measurement as inferred from the Cho measurement (inverse [Cho]), from both the control and the HC-3 reactions. Asterisks are shown in places where a calculation could not be made due to limitations in the sensitivity of the MBD method. Bars are shown where measurements were not made.

	Control Reaction (μM)					HC-3 Reaction (μM)		
Minutes	[PCho]	Inverse [Cho]	[Cho]	[Cho] MS	[PCho] MS	[Pcholine]	[Cho] MS	[Pcho] MS
0	*	72.6	927.4	-	-	*	-	-
20	284.3	270.4	729.6	-	-	*	-	-
40	374.8	367.7	632.3	-	-	258.5	-	-
60	451.7	445.2	554.8	521.1	422.2	284.9	741.1	295.2
80	514.4	502.5	497.5	478.7	540.9	325.5	693.1	326.2
100	551.7	579.2	420.8	462.6	549.5	354.3	668.9	375.3

**Table 2:** Precursor-product ion transitions.

Name	Precursor *m*/*z*	Product *m*/*z*
Choline	104	45
Phosphocholine	184	86
